# Management of Acute Stroke in the Older Person

**DOI:** 10.3390/geriatrics2030027

**Published:** 2017-08-15

**Authors:** Emma Parr, Phillip Ferdinand, Christine Roffe

**Affiliations:** 1 Keele University Medical School, Newcastle Road, Stroke-on-Tent ST4 6QG, UK; v5y18@students.keele.ac.uk; 2 Stroke Research in Stoke, University Hospitals of North Midlands NHS Trust, Newcastle Road, Stoke-on-Trent ST4 6QG, UK; phillip.ferdinand@nhs.net; 3 Faculty of Medicine and Health Sciences, Guy Hilton Building, Thornburrow Drive, Stoke-on-Trent ST4 7QB, UK

**Keywords:** acute stroke, treatment, elderly

## Abstract

The majority of people who suffer a stroke are older adults. The last two decades have brought major progress in the diagnosis and management of stroke, which has led to significant reductions in mortality, long-term disability, and the need for institutional care. However, acute, interventional and preventative treatments have mostly been trialled in younger age groups. In this article we will provide an overview of the evidence for acute stroke treatments in relation to age, discuss special considerations in the older person, and contemplate patient choice, quality of life, and end-of-life-decisions.

## 1. Introduction

Stroke is the third leading cause of life years lost worldwide [1], and is the third most common cause of disability [2]. Acute ischaemic stroke accounts for 85% of all strokes whilst the remaining 15% is attributable to haemorrhage. The last two decades have seen major advances in the diagnosis and management of stroke, resulting in a significant decline in the age standardized incidence, mortality and disability adjusted life years between 1990 and 2013. The mortality from ischaemic stroke in developed countries has almost halved (112.9/100,000 to 67.2/100,000) in the same period [3]. However, the absolute number of people affected by stroke worldwide has increased, with a 19% rise in the global burden of disease between 1990–2010 [2].

This increase in stroke burden is attributed to a combination of population growth and an increasing ageing population [2]. Age is one of the non-modifiable risk factors for stroke, and there is therefore a correlation between the increasing incidence of stroke and the growing number of older people within the population, which poses predictable challenges for health and social care systems. In addition, people aged over 80 have an increased risk of frailty and multiple co-morbidities, and are also more likely to have a severe stroke, which makes their management more complex [4]. The third challenge associated with this increased prevalence of stroke in the older population is that the very old are often excluded from clinical trials, and therefore there is little evidence of whether treatments are as effective as they are for those under 80 years of age [5,6].

In this article, we will review the evidence for stroke treatments in relation to age, discuss special considerations in the older person, and contemplate patient choice, quality of life, and end-of-life-decisions.

## 2. Hyperacute Stroke Treatments

Hyperacute stroke treatments are aimed at preventing brain damage by reversing the cause of the stroke (unblocking occluded arteries, halting brain haemorrhage, improving brain perfusion) and preventing stroke progression. These have to be given as soon as possible after stroke onset, and before permanent brain damage is established. This is usually within the first nine hours of stroke onset.

### 2.1. Care on Specialized Stroke Units

Over the last decade, there has been a paradigm shift in the management of stroke patients, involving the transfer of care from general wards into dedicated multidisciplinary stroke units. The idea of specialized stroke units was first conceptualized in the 1980s, but only widely implemented after meta-analyses of multiple small trials showed that organized stroke care led to significant improvements in outcome, with a 20% reduction in both mortality alone (odds ratio (OR) 0.81) and the combined outcome of death and disability (OR 0.79). These improvements were observed both in a mixed population of strokes [7], and also in patients with intracerebral haemorrhage as the presenting problem [8]. It is of particular importance for older people, who often fear having to move into a nursing home, that stroke-unit care also significantly reduces the risk of new institutionalization, irrespective of age (OR 0.77, 95% CI 0.63 to 0.94 for age < 75 and 0.69, 95% CI 0.56 to 0.85 for age > 75 years), sex, or initial stroke severity [7]. Stroke-unit care improves outcomes due to better prevention of complications. It is therefore important that all stroke patients, including the very old, are admitted to specialist care as soon as possible.

### 2.2. Thrombolysis

Since the publication the National Institute of Neurological Diseases Stroke (NINDS) trial in 1995 [9], intravenous thrombolysis using alteplase has become an important part of hyperacute stroke treatment. The initial licence was restricted to patients under the age of 80 years, as older patients were excluded from trials assessing thrombolysis. It was not until the Third International Stroke Trial (IST-3)—the largest study of thrombolysis in acute stroke to date—that patients over the age of 80 were included. Fifty-three percent of the 3035 patients enrolled in the study were over the age of 80 [10,11]. An early increase in mortality due to intracerebral haemorrhage was counterbalanced by later reductions in death rates, so that overall mortality at 6 and 18 months was not affected by thrombolysis [10,11]. The results confirmed that “time is brain”, with best outcomes in patients treated in less than three hours from symptom onset. Benefit from treatment was greater in those aged over 80 than in younger subjects (*p* = 0.029 for the interaction term). A meta-analysis (heavily weighted by IST-3 data with an additional 69 over 80-year-olds included from NINDS [9], and 25 from the Effects of alteplase beyond 3 h after stroke in the Echoplanar Imaging Thrombolytic Evaluation Trial EPITHET [12]) of all acute thrombolysis randomized controlled data confirmed the benefit in over 80-year-olds, with 96 more patients surviving and independent for every 1000 treated within three hours [13]. A further meta-analysis of the same data in 2014 confirmed the finding that alteplase significantly improves the overall odds of a good stroke outcome when delivered within 4.5 h of stroke onset, irrespective of age or stroke severity, despite an increased risk of fatal intracranial haemorrhage during the first few days after treatment [14].

There are no data on outcomes in the very old (>90 years old) from randomized controlled trials. IST-3 included 210 patients over the age of 90, but did not analyze this group separately. The largest observational study recruited patients from the US ‘Get with the Guidelines’ programme. Out of 35,708 patients who presented within two hours of stroke onset, 2895 (7.2%) were >90 years old. Of these, 67% were thrombolysed. Rates of symptomatic haemorrhage were similar to younger patients (6%). Good functional outcome (14% discharged home) was lower, and mortality (36%) higher than in younger patients, as expected in this older and frailer population [15].

The elderly remain a particularly heterogeneous group in terms of co-morbidity and dependency. While thrombolysis clearly shows a benefit in fit and independent older patients who meet the criteria to participate in clinical trials, many elderly patients have significant physical and cognitive co-morbidities, and the risk/benefit ratio for thrombolysis is less well defined. None of the thrombolysis trials included patients who were dependent before the stroke. However, even small benefits, such as regaining the ability to speak or swallow, can make a big difference in quality of life in this patient group. In the very old and very frail the decision to thrombolyse should be made on an individual patient basis.

### 2.3. Mechanical Thrombectomy

The advent of techniques to extract clots from major cerebral vessels, coupled with the increased availability of advanced imaging, have allowed for a more accurate selection of suitable patients, and led to a recent string of positive trials for endovascular treatment in acute stroke. Three out of six of these trials had no upper age limit (MR CLEAN, EXTEND IA and ESCAPE) [16,17,18]. The primary outcome in all trials was the modified Rankin Scale (mRS) score at 90 days. MR CLEAN reported a significant shift in the distribution of mRS scores in favour of thrombectomy (adjusted common OR 1.7). Eighty-one of the 500 patients included were over 80 years old, and the sub-group analysis by age showed a greater likelihood of benefit in the over-80s (OR 3.2) than in the under-80s (OR 1.6). In ESCAPE the common OR for a shift to a better mRS was 2.6 in favour of endovascular intervention, with similar benefit for the under 80s (OR 2.7) and the over-80s (OR 3.0). Sub-group analysis by age was not performed in EXTEND IA, as only 70 patients were enrolled due to the early closure prompted by the results of MR CLEAN.

The remaining three trials (SWIFT-PRIME, REVASCAT and THRACE) [19,20,21] performed subgroup analyses by age using 70 years as the cut off. In SWIFT-PRIME, the relative risk of functional independence (mRS 0–2) at 90 days was 1.67 and 1.75 for the under-70s and over-70s respectively. In THRACE, benefit from thrombectomy was also similar in the younger and older subgroups (OR 1.58 and 1.54 respectively for the same age cut offs), while in REVASCAT, benefit was only seen in the under-70s (OR 2.5), but not in the older age group (OR 0.9). The reasons for this are not clear, as the characteristics of the sub-group were not presented. The subsequent HERMES collaboration (pooling of data by investigators of MR CLEAN, ESCAPE, EXTEND IA, SWIFT-PRIME and REVASCAT) found that the common OR for a favourable independent outcome was 3.68 (1.95–6.92) in the 80-and-over age group (198 patients), and 2.41 (1.55–3.74) in the 70–79 sub-group, which was greater than, and comparable to, the other age sub-groups, respectively [22].

Whilst the effect of endovascular treatment may be exaggerated due to the small numbers in the over-80s sub-group and selection of physically fit individuals, it does nonetheless suggest that the oldest patients may have at least as much to gain from this procedure as younger people, and that age alone should not be a factor when making the decision to offer thrombectomy to such patients.

### 2.4. Decompressive Hemicraniectomy

Following a large infarct tissue necrosis leads to oedema, and this can cause herniation of the brain and early death [23]. The only effective treatment is decompressive hemicraniectomy, which involves the temporary removal of part of the skull to relieve pressure and prevent herniation. This has to be performed within 48 h of stroke onset. The procedure is indicated if the neurological deficit is severe (National Institutes for Health Stroke Scale score >15) and computed tomography (CT) shows an infarction in 50% of the middle cerebral artery territory or an infarct volume of >145 cm^3^. [24]. In patients up to the age of 60 years decompressive hemicraniectomy increases one-year survival from 29% to 78%, with the number needed to treat to save one life being two. Half of the survivors were able to walk, but needed help with activities of daily living (mRS 3), and the other half had more severe disability (mRS 4–5). The number needed to treat for one patient to survive with mRS ≤3 was four [25,26]. Older patients were excluded from the first trials of decompressive hemicraniectomy, as the risk of herniation is lower due to age-associated brain atrophy, and also because the procedure was considered too invasive for this age group. [25,26] A more recent study, DESTINY-2, included patients over the age of 60 and demonstrated a survival benefit up to the age of 82 years, with the number needed to treat to save one life being four. As in younger patients, all survivors had moderate to severe disability and were dependent on care (10% mRS = 3, 57% mRS = 4, and 34% mRS = 5) [27].

After one year quality of life was the same in all survivors, irrespective of whether they had a craniotomy or not. Sixty-three percent of survivors in the craniotomy group, and 53% of surviving controls agreed when asked, “Do you, in retrospect, consent to the treatment you received?” Compared to persons in the same age range who have not had a stroke, most domains of quality of life on the SF 36 tool (mental, emotional, social function, vitality, pain, health) were similar in these survivors, except for motor function, which was lower [27]. When discussing risks and benefits of the procedure with patients or their families, it is important to convey information on mortality, disability, and quality of life to enable them to make a balanced and informed decision.

## 3. Acute Stroke Care

Whilst the purpose of hyperacute stroke care is to prevent brain damage, acute stroke care is focussed on the prevention of complications and providing the best chance of recovery. The key evidence for the effectiveness of stroke units is derived from studies conducted before hyperacute interventions such as thrombolysis, thrombectomy and craniotomy were introduced. More deaths after stroke are due to complications, such as pneumonia, than due to neurological damage. Prevention and effective treatment of complications are therefore crucial for reducing mortality after stroke. This is particularly important in older stroke patients, as they are at greater risk of developing complications. While there is clear evidence that specialist stroke unit care as a whole is effective, considerably less is known about the effectiveness of individual interventions, especially in relation to age.

### 3.1. Dysphagia

Dysphagia (difficulty swallowing associated with foods, fluids and saliva) is a common complication of stroke, with about 50% of patients affected acutely [28]. The prevalence varies widely, ranging from 28% to 78%, depending on time and type of assessment [29,30,31]. The risk of post-stroke dysphagia increases with age [28,32] due to pre-existing swallowing problems [33] and reduced functional reserve [34]. There is a correlation between swallowing stages and the location of the brain injury [35]. Swallowing problems are related to the size of the swallowing cortical area in the unaffected hemisphere, which may reflect the capacity for compensation [36]. Dysphagia is a major risk factor for aspiration pneumonia [31,37] and an independent predictor of death and institutionalization [28].

In most stroke patients swallowing function improves spontaneously in the first few days [31], partly due to bilateral cortical representation of neurological pathways [36]. However, 13% of patients are left with persistent dysphagia [38], with attendant risks of malnutrition and dehydration [29]. Just as importantly, dysphagia can make affected individuals avoid eating in social settings, which deprives them of the physical and social pleasures connected with food and drink [39].

Formal screening for dysphagia reduces the risk of pneumonia by 60%, and has a major impact on outcomes [40]. Swallowing problems are managed by modification of diet and fluids, and, if this does not suffice to reduce the risk of aspiration, by replacement of oral intake with enteral nutrition and hydration [30,33]. However, while enteral feeding prevents starvation, it does not prevent pneumonia [41]. As nasogastric feeding is associated with a significant risk of pneumonia, and the risk of pneumonia is highest in the first week after the stroke, the FOOD trial compared early nasogastric feeding with delaying feeding for at least a week, and demonstrated that early feeding was safe and associated with a trend towards lower mortality (n = 859, median age 78). While nasogastric feeding is often insufficient to meet nutritional demands because the tube easily gets displaced, which interrupts the feed, the study also showed that changing early from nasogastric to feeding via a percutaneous gastrostomy was associated with higher mortality, a higher institutionalization rate, and a lower probability of returning to normal oral feeding (n=32) [42]. Uncertainty remains about how to rehabilitate swallowing. Postural and compensatory techniques are sometimes used to improve safety, but this has not been tested in clinical trials. There is emergent evidence for functional electrical and pharyngeal stimulation, but more research is needed to determine which interventions are effective, and when [30].

### 3.2. Venous Thromboembolism

Venous thromboembolism [VTE] is a common, potentially avoidable cause of morbidity and mortality in hospitalized patients, and specifically in patients who have had a stroke, with rates up to 50% by direct thrombus imaging [43]. The incidence of symptomatic thromboembolism is considerably lower at 3% for deep vein thrombosis (DVT), and 1% for pulmonary embolism (PE). DVT can cause limb oedema, pain, tenderness, and fever, although it is more frequently asymptomatic (7% of strokes) [44]. Fatal pulmonary embolism is estimated to occur in about 15% of patients with untreated proximal DVT [45]. Most DVTs develop early, within the first week after the stroke. Old age, severity of paralysis, and dehydration are important risk factors. Patients above the age of 70 are four to six times more likely to develop a DVT or PE than younger patients [46], and this risk doubles with each decade [47]. Venous thromboembolism is more likely to be fatal in older patients (OR for age >75 years 3.18) [48], due to the direct effects of the embolism and to comorbidities. The diagnosis of VTE in the elderly is challenging, as an atypical presentation and a reduced sensitivity and specificity of both clinical scoring systems and laboratory parameters impede a timely diagnosis.

There is strong evidence from large clinical trials and meta-analyses that prophylactic anticoagulation with heparin (unfractionated or low molecular weight) is effective at preventing DVT, but also increases the risk of both intracranial and extracranial haemorrhage. A meta-analysis examining risks and benefits of prophylactic anticoagulation has shown that prevention of symptomatic PE was counterbalanced by an increase of symptomatic intracranial haemorrhage [49]. Based on this evidence, prophylactic anticoagulation is not recommended for unselected stroke patients in the UK stroke guidelines [50]. Results of the CLOTS-1 and -2 studies showed that graduated compression stockings (either full-length or below-knee) are not effective in preventing VTE in stroke patients [51]. Evidence from the CLOTS-3 study shows that intermittent pneumatic compression (IPC) not only reduces DVT, but also mortality. There was no sub-group analysis according to age. This treatment should be offered within three days of hospitalization and be provided continuously for 30 days, or until the patient is mobile or discharged, whichever is sooner [52]. However, once a PE or symptomatic DVT is diagnosed in patients with ischaemic stroke, full therapeutic anticoagulation is recommended [50]. In patients with an intracerebral haemorrhage, a vena cava filter might be an alternative [53], but this also requires anticoagulation in the longer term.

### 3.3. Decubital Ulcers

The term refers to a wound that develops in the upper layers of the skin, then enlarges both radially and into the deeper tissue layers. The primary risk factor is prolonged pressure on a particular part of the skin. Differing sensitivity of individuals to tissue damage depends on the balance between externally applied pressure and capillary pressure in the tissue layers underneath, which is determined by blood pressure and the presence of peripheral arterial occlusive disease. Shear stress, friction and moisture further contribute [54]. There are national and international evidence-based guidelines for the prevention of pressure ulcers [55,56]. Recommendations are split into those for adults and children/neonates without specific recommendations for the older person.

Immobility [57], malnutrition, dehydration, urinary and faecal incontinence all increase the risk of pressure ulcers [58]. Stroke patients are particularly at risk due to hemiparesis, which reduces their ability to change position; hemianaesthesia and hemiinattention, which affect the ability to perceive detrimental pressure; and dysphasia and confusion, which prevent effective communication of pain. Elderly stroke patients are at an even higher risk due to increased skin fragility, which is part of the ageing process [59]. A variety of screening tools (e.g., Braden, Norton, and Waterlow scales) have been developed to identify high-risk groups [60]. These are completed by nurses on admission and guide care procedures and choice of mattress.

Key aspects for prevention of pressure sores in adults recommended by the National Institute for Health and Care Excellence (NICE) are risk assessment, skin assessment, regular repositioning (at least four-hourly for individuals at high risk), the use of mattresses appropriate for the level of risk, pressure redistribution devices, and barrier creams. Skin massage for the prevention of pressure ulcers is discouraged, and nutritional supplements are only recommended if there is malnutrition.

In patients with stroke, the sacrum, buttocks, and heels are the usual sites for pressure sores and should be examined frequently. Early mobilization, regular change of position, heel protection devices, the use of appropriate pressure-relieving mattresses [29], the effective treatment of diarrhoea, and the avoidance of dehydration, malnutrition, and hypotension, are particularly important in this group. Appropriate handling and the use of glide sheets are important to reduce shear stress and secondary skin damage when repositioning patients in bed. When seated, pressure-relieving pads can be used to prevent skin damage. It is also important to ensure that both feet are in contact with the ground, as this allows the patient to change position and reduce pressure on the sacrum.

### 3.4. Incontinence

Forty to fifty percent of stroke patients are incontinent in the acute phase [61,62,63,64]. The prevalence falls to 19% at three months, and to 15% at one year [65]. Elderly patients with stroke often have pre-existing difficulties with micturition, but continence problems can also develop as a new complication following a stroke. Urinary incontinence is most common in patients with severe strokes [66,67].

The impact of urinary incontinence on patients’ recovery following a stroke is substantial, with high rates of disability, mortality and institutional care [68]. Stroke patients have a higher risk of developing a urinary tract infection (UTI) than non-stroke patients, with symptoms on average developing 17 days post-stroke. This may be due to immunosuppression, bladder dysfunction, or increased likelihood of catheter placement post-stroke. The risk of UTI is particularly high in older stroke patients [69]. Incontinence has a detrimental effect on mood, confidence, self-image, and the ability to participate in rehabilitation [50].

The causes of incontinence after stroke include urge, stress, and environmental factors such as immobility and disorientation, with more than one contributing in most stroke patients. Evidence for the effectiveness of individual interventions for treatment of incontinence after stroke remains poor. A Cochrane systematic review in 2008 identified several treatment options. There was some evidence that specialist nurse input improves both incontinence and patient satisfaction. There were only three small studies of pharmacotherapy. One, testing meclofenoxate, showed a reduction in incontinence, while there was no difference with oxybutynin or oestrogen treatment. Studies of behavioural interventions (systematic voiding, pelvic floor exercises) were too small, and a study of complementary therapies (acupuncture) was not of sufficient quality to test effectiveness [64]. Since then, it has been shown that the introduction of a systematic nurse-led voiding programme is feasible in the NHS setting [70], but that there are barriers to implementation, which might be overcome by staff training, the involvement of patients and carers, increased staffing levels [71]. There is also emerging evidence from a small study to suggest that transcutaneous electric nerve stimulation in the lumbar area has some effect on nocturia, frequency and urgency [72]. Electroacupuncture has shown some improvement in very young stroke patients with urge incontinence (mean age 39) [73]. Further, more recent, small trials suggest that electroacupuncture [73], pelvic floor exercises [74,75], and moxibustion [74] may be effective in the treatment of urinary symptoms [76]. There is a clear need for adequately powered trials to test promising interventions further.

The UK stroke guidelines suggest that stroke unit staff should be trained in the use of standardized assessment and management protocols for urinary incontinence, and that indwelling catheters should not be used unless necessary to relieve retention or asses fluid balance [50]. Treatment plans for patients with incontinence lasting greater than two weeks should include more detailed reassessments to identify the cause, behavioural interventions (e.g., timed toileting, prompted voiding, bladder retraining, pelvic floor exercises), training of the patient and carer, arrangements for the supply of continence aids, and referral to specialist services, with pharmaceutical treatments and long-term catheter as potential options if all else fails [50].

### 3.5. Urinary Retention

Urinary retention is a common complication of acute stroke. It can occur immediately after the stroke or later, and affects both men and women. Causes include pre-existing problems (prostatic enlargement), medications (anticholinergics, diuretics), detrusor areflexia, constipation, immobility (inability to stand for urination, or sit on the toilet), and inadvertent discontinuation of medications such as alpha-1 receptor antagonists [77]. Urinary catheterization may be necessary to relive acute retention, but is also associated with significant risks. Catheter-associated UTI is the most common nosocomial infection in hospitals. The longer a catheter is in place, the greater the danger. One to four percent of patients with a catheter-associated UTI develop bacteraemia, with a mortality of 13–30% [78]. Catheter use should be intermittent in the first instance, with longer-term catheterization restricted to patients where precipitating factors cannot be addressed [77].

While most older men with retention after stroke have some degree of prostatic enlargement, this alone is not the cause of retention in patients who were able to pass urine normally before the stroke. Patients with urinary outflow obstruction can only micturate when the bladder has a filling volume high enough to support high detrusor pressures and overcome the obstruction. If the patient is not toileted at that point in time, bladder volume increases further, leading to the overstretching and weakening of the detrusor muscle. Under these conditions, retention can be caused by the inability to go to the toilet at the time point when the bladder has the optimal filling pressure. Frequent toileting and/or intermittent catheterization can overcome this problem, and allow the patient to return to their normal bladder habits. The HOUDINI guidelines provide a clear pathway for catheter removal, determined by underlying pathology and residual volume on bladder scan [79].

While intermittent catheterization after stroke is only a temporary measure to allow bladder recovery, it can also be considered in patients with permanent outflow obstruction. This method is usually recommended in younger patients, but it has also been shown to be feasible and acceptable in older people [80].

### 3.6. Falls

Falls are a common complication of stroke and can have significant adverse effects on rehabilitation. In the acute phase of stroke (the first 10 days), 8.4% of patients experience falls (1% serious, 7.4% non-serious) [81]. At this stage the major determinant of falls is stroke severity, with most occurring in moderate strokes (18%), and considerably fewer in very mild and very severe strokes (3% respectively) [82]. Falling is a common complication throughout all phases of stroke recovery [83]. With a two-year incidence of 46%, 15%, and 2.1% observed here in a longitudinal study of a large nationwide cohort, falls, fall-related injuries, and hip fractures represent serious long-term complications for stroke survivors [84]. In severe strokes (Barthel Score <10/20), the risk of falls is even higher (46% at three months, 76% at one year) [57]. Falls are attributable to deficits in balance, muscle weakness, gait deficits, the impairment of cognitive function, reduced attention, and abnormalities in vision, but are not generally associated with age [85,86]. Complications of falls include hip fractures, functional limitations, and development of a fear of falling. The latter can be more disabling than actual injuries, with limitations to mobility imposed by self or others leading to deconditioning, social isolation and loss of independence [83,87].

There is little to no evidence of effective interventions to prevent falls in hospital. For older people in the community, a Cochrane review highlighted the effectiveness of multifactorial programmes, such as exercise for patients aged over 75; however, this is not directly translatable to patients with stroke [83,88].

UK Stroke guidelines stress the importance of rigorous physiotherapy assessments to reduce the risk of falls. Interventions to prevent falls include education and adaptations e.g., low bed, chair alarms. These interventions are often multifactorial, addressing physical and psychological aspects. People with stroke who have symptoms of vitamin D deficiency, and those who are considered to be at high risk (e.g., housebound) should be offered calcium and vitamin D supplements [50].

As there is no evidence that imposed restriction of mobility prevents falls or injury, and quality of life of stroke survivors is affected by fear of falls, it is important to encourage continued mobility and provide advice on how to get up from the floor after a fall.

### 3.7. Delirium

Delirium is characterized by the acute onset of an altered level of consciousness with a fluctuating course affecting orientation, thought, or behaviour. It usually is self-limiting, and most patients recover completely within a week.

A systematic review and meta-analysis including 10 studies and more than 2000 stroke patients, found that the incidence of post-stroke delirium ranged from 10% to 28% [32,89]. Individuals who developed delirium stayed nine days longer in hospital, were three times as likely to be discharged to a long-term care facility, and had a fivefold increase in mortality [90]. Delirium can develop any time following a stroke, and is often caused by medication, infection, heart failure, metabolic abnormalities, or hypoxia [89]. Predisposing factors for delirium include urinary or respiratory infections and pre-existing cognitive impairment, and all of these are more prevalent in the elderly [89]. Post-stroke delirium can be predicted with a sensitivity of 76% and specificity of 81% using a tool based upon age, stroke severity, subtype and infection [91].

Up to one third of episodes of delirium can be avoided by a multicomponent intervention targeting cognitive impairment, sleep deprivation, immobility, visual and hearing impairment, and dehydration [92]. It is important to provide the right environment to limit confusion. Once established, identification and treatment of underlying causes of delirium is the most important and effective management strategy. Sedative and antipsychotic medications should be avoided, especially in those with a history of dementia, because of an increased risk of death (haloperidol, olanzapine, quetiapine, risperidone), stroke (olanzapine) [93,94,95,96], and falls [97]. Recent studies have explored the possibility of using melatonin agonists and rivastigmine for delirium after acute stroke in the elderly [91].

## 4. Special Considerations

### 4.1. Do Not Attempt Resuscitation Orders

In most stroke patients cardiac arrest is the final event and expected as a consequence of severe neurological injury, pneumonia, or other complications of the stroke. As these pathologies progress over time, appropriateness of resuscitation can be considered as death approaches, with ‘do not attempt resuscitation’ (DNAR) orders preventing futile resuscitation. However, stroke patients are also at increased risk of sudden and unexpected death due to pulmonary embolism, metabolic disturbances, and myocardial infarction. Therefore, during hospitalization, it is important to address the wishes of the patient with regard to cardiopulmonary resuscitation (CPR) [98]. When discussing resuscitation, it is essential to give as accurate as possible information relating to the prognosis for survival and disability, and to consider burdens associated with the proposed treatment [99]. It is also important to be aware of the ‘disability paradox’, which describes the phenomenon that many people with serious and persistent disabilities report they experience good quality of life when most external observers would consider their symptoms unbearable [100,101]. Where death is inevitable, it is no longer appropriate to discuss resuscitation, but conversations with the patients and their families should focus on communicating the proximity of death, and on their needs and wishes. While DNAR orders should not affect other aspects of care, there is evidence that early DNAR is associated with increased mortality, even when adjusted for other comorbid factors [102,103,104,105,106]. When discussing DNAR, these risks should be considered, along with the potential burdens of the procedure and the effects of survival with disability.

### 4.2. Priorities

When choosing end points for clinical trials, views on what is important can differ between researchers and patients. In a study of 785 cardiovascular patients and 164 clinical trial authors, the relative importance of individual end points (e.g., death, myocardial infarction, stroke, coronary revascularization, and hospitalization for angina) within a composite outcome were weighted. Whereas patients assigned similar weights to death, myocardial infarction, and stroke, clinical triallists were much more concerned about death. Both patients and triallists considered revascularization and hospitalization as substantially less severe than death. With increasing age, the importance of death declined in favour of myocardial infarction and stroke. This was particularly evident over the age of 85, where stroke was weighted as most important [107].

Knowing patients’ priorities is particularly important when making decisions in relation to treatments that might improve quality of life, but risk death. A study of older patients’ views on taking part in a trial of thrombolysis suggests that their interpretation of risk may be different compared to younger people. Responses included statements such as: "Four people in 100 is a very small risk compared to living a vegetable life”, "I think at my age I have nothing to lose", and "The quality of life is what matters”. One of the focus groups thought a maximum average risk of up to 20% of immediate death was acceptable [108].

When patients’ priorities differ significantly from those of the professionals looking after them, it is often assumed that they are not able to assess the risk appropriately. While this is a definite possibility in stroke patients who have significant brain injury, it is also important to consider that their preferences might differ because of their age and personal circumstances.

### 4.3. End of Life

High quality end-of-life care is a core activity for any multidisciplinary stroke team. Such high quality care is described as holistic, delivered by trained individuals in the best interest of patients, and with advice from the palliative care team. These recommendations are mostly based on evidence from cancer patients, as there are only few good quality end-of life studies in stroke patients [50]. Key features of palliative care in stroke patients are summarized in a statement for healthcare professionals from the American Stroke Association: care should be patient- and family-centred, and the responsible clinician and their team should estimate prognosis accurately, develop appropriate goals, be familiar with the implications of end-of-life decisions, assess and manage symptoms effectively, assist with care coordination, provide the patient and the family with opportunities for personal growth, and participate in continuous quality improvement and research [98].

Decisions about end-of- life care should ideally involve the patient as a key decision maker. In patients who have had a stroke severe enough to consider palliative care, this is rarely possible. However, in many cases, at least some preferences can be determined by observing facial expressions and gestures. Questions like ‘Are you hungry/thirsty/hot/in pain’ should be attempted wherever possible. Enquires worded as ‘Are you happy for me to do this? Is this treatment bothering you? Do you want me to stop doing this?’ can also be helpful. Answers need to be retested for consistency. Including a few nonsense questions can help to gage reliability of the yes/no answers.

Conversations with family and friends about pre-stroke personality, wishes, likes and dislikes, as well as observations of behaviours and moods, and advance directives should all be taken into account when making decisions for patients. Such discussions will inevitably centre on balancing quality with quantity of life. When trying to gain such information from surrogate decision makers, it is important to consider that patients with disabilities are likely to rate their quality of life higher than healthy people who are asked to imagine themselves in the same situation [109]. This may be due to a tendency to concentrate on the disabilities, rather than on remaining abilities. It is also important to realize that patients can adapt to disabilities, even those previously considered unimaginable [101].

Palliative care in stroke patients differs in several respects from that in other terminal illnesses. Compared with other palliative conditions, patients with stroke are more physically impaired and less able to express their needs. They are more likely to die in hospital, and less likely to suffer from severe pain. While different, palliative care needs of both the patient and their families are often enormous.

## 5. Conclusions

Acute stroke care has changed greatly over the last two decades, driven by evidence from clinical studies and systematic reviews. Significant reductions in mortality were achieved by the systematic introduction of specialized stroke unit care. Improvements in functional recovery are due to hyperacute stroke treatments that reduce permanent brain damage, and the prevention of complications on acute stroke units, which allows for better recovery. While there is strong evidence to support hyperacute treatments such as thrombolysis, thrombectomy, admission to specialist stroke units, and decompressive hemicraniectomy, much less is known about the best way to prevent complications and promote recovery. There is a need for research focussing on the implementation of effective acute stroke treatments, prevention and treatment of complications, and approaches to rehabilitation therapy. Where evidence for effectiveness in relation to age exists, it does not support the restriction of active management to younger age groups. Decisions about offering invasive and potentially burdensome treatment, and, conversely, about the limitations of active management and palliative care, are difficult. Such decisions should be guided by evidence and the wishes of the patient, or, where this is not possible, by those who know their preferences best. More research is needed to inform clinical care and decision making in relation to patient preferences at the end of life.
